# Effect of thermoplastic polyurethane filament on the cellular ceramics structures obtained from material extrusion and polymer-derived ceramic

**DOI:** 10.1007/s40964-025-01243-w

**Published:** 2025-07-23

**Authors:** Apoorv Kulkarni, Louisa Eckey, Pietro Mosca, Rajat Chaudhary, Amir Hadian, Joshua M. Pearce, Frank Clemens, Gian Domenico Soraru

**Affiliations:** 1 Western University, London, Canada; 2 Empa - Swiss Federal Laboratories for Materials Science and Technology, Dübendorf, Switzerland; 3 University of Trento, Povo, Trento, Italy

**Keywords:** Material extrusion, Fused filament fabrication, Polymer-derived ceramics, Thermoplastic polyurethane, Silicon oxycarbide, Polysilazane

## Abstract

Cellular ceramic structures were fabricated via 3D printing of thermoplastic polyurethane (TPU) followed by impregnation with polysilazane, and pyrolysis. The 3D printing was performed using fused filament fabrication (FFF), while the ceramic was obtained through the polymer derived ceramic (PDC) process starting from a commercially available polysilazane, Durazane 1800. We investigated the role of ester- and ether-based TPUs with two different Shore hardness (90A vs 80A) on the impregnation of polysilazane. Regardless of the TPU type and Shore hardness, impregnation of the TPU 3D structure was successful and resulted in dense, non-hollow ceramic struts after pyrolysis. All polyester- and polyether-based TPUs showed a similar mass and volume increase after impregnation with high deviation. The mass loss during pyrolysis was also very similar for all the TPUs. The behavior of these TPUs was then compared with one commercial TPU filament (Ninjaflex with a Shore hardness of 85A). While the Ninjaflex 3D-printed structures showed a greater increase in mass and volume after impregnation, the pyrolysis outcome was almost identical to that of the samples fabricated with both ester- and ether-based TPUs, resulting in dense, non-hollow ceramic struts.

**Supplementary Information:**

The online version contains supplementary material available at 10.1007/s40964-025-01243-w.

## Introduction

Additive manufacturing (AM) or 3D printing of ceramics has made significant progress in the last decade [1, 2]. Ceramics can be manufactured with AM techniques, such as fused filament fabrication/fused deposition modeling (FFF/FDM) [3, 4], selective laser sintering (SLS) [5, 6], stereolithography (SLA) [7], laminated object manufacturing (LOM) [8, 9], binder jetting [10], directed energy deposition [11], extrusion-based printing like robocasting [12], and direct ink writing (DIW) [13].

Cellular ceramics, mainly processed by AM, have been the center of attention as they can be designed in a geometry with a high degree of complexity. Combined with their excellent thermal and mechanical properties, they find use in various fields [14, 15], such as liquid metal filtration for castings [16], and gas and chemical filtration [17, 18]. Cellular ceramics have also found importance in biotechnology when used as scaffolds for bone regeneration or drug delivery [19–22]. Other applications of cellular ceramics include heat exchangers [23] and support for catalysis [24]. A recent review papers highlights the novel processing techniques (including AM) and application of porous ceramics [25, 26]

Polymer derived ceramics (PDC) [27–29] have further facilitated 3D printing of silicon-based cellular ceramic structures [30, 31]. PDCs use Si-containing preceramic polymers that, upon pyrolysis at high temperature in a controlled (inert or reactive) atmosphere, lead to ceramics of the general composition Si–O–C–N. Researchers have successfully showcased the production of cellular structures using PDCs. These methods are useful to shape the preceramic polymers as the feedstock by AM. For example, Zanchetta et al. [32] used a liquid polysiloxane mixed with photoinitiators as resin for stereolithography. The produced green parts were pyrolyzed to obtain SiOC cellular structures. Similarly, other researchers have used ink solutions composed of polysiloxanes and polycarbosilanes to 3D print cellular structures and then convert them into silicon based ceramic structures [33].

Another method to produce cellular ceramics uses sacrificial scaffolds. Polymeric cellular structures such as foams can be coated with a ceramic slurry or impregnated with preceramic polymer solution [15, 16]. After pyrolysis the polymeric structure decomposes completely, leaving a self-similar cellular ceramic component.

With the advances in 3D printing, researchers have demonstrated carefully designed 3D-printed cellular structures that can be used as sacrificial scaffolds instead of foams. This fabrication method gives us better control over the size and shape of the component as well as the porosity of the structures [34, 35].

In principle, there are two types of porous structures that can be obtained with the replica method using preceramic polymers. One is a hierarchical porous structure, where the preceramic polymer just coats the sacrificial polymeric structure. This allows the processing of cellular ceramics with hollow struts [35]. The other type has dense struts, where preceramic polymer solution diffuses into the core of the FFF 3D-printed sacrificial polymeric struts [36]. During pyrolysis, when the organic polymer decomposes, it leaves behind a dense ceramic strut resulting from the organic-to-inorganic transformation of the preceramic polymer.

This process relies on the mutual solubility (compatibility) of the Si-preceramic polymer with the organic polymer used for the FFF. So far, it was demonstrated that commercial thermoplastic polyurethane (TPU) (NinjaFlex, Fenner Precision Polymers, Lititz, PA, USA) and a commercial polysilazane, Durazane 1800, CAS: 503590-70-3; Merck, Darmstadt, Germany) combination can be used to achieve 3D-printed cellular structures with dense struts [36]. After immersing the FFF 3D printed TPU cellular structure in the polysilazane solution, the confirmation of compatibility between these two polymers is evident through the permanent structure's swelling (increase in volume). A higher relative volume and mass increase signifies greater diffusion of preceramic polymer within the template, leading to increased ceramic yield and improved quality of the resulting ceramic structure.

This study focused on analyzing two types of thermoplastic polyurethanes (TPUs), specifically polyesterol- and polyetherol-based variants with varying shore hardness, in addition to a previously studied commercial TPU. Our primary objectives included filament production, 3D printing, the impregnation process, and determining ceramic yields post-pyrolysis. To ensure experimental consistency, we utilized the same preceramic polymer (Durazane 1800) as in earlier research. These cellular ceramic structures could find application in different fields such as scaffold for bone regeneration [22] or high temperature components like heat exchangers of catalyst supports [37].

## Materials and methods

### Manufacturing of TPU filament

The polyester- and polyether-based TPU pellets, namely, Elastollan 90 A, 1190, 80 A, 1180 (BASF, Ludwigsburg, Germany) with two different shore hardness (SH) 90 A and 80 A were extruded to filaments using a capillary rheometer (Rosand, RH7 Netzscch GmbH, Selb, Germany), respectively (see Table 1). The pellets were dried before extrusion for 24 h to 80 °C. An extrusion speed of 10 mm/s, at 185 °C, was applied to achieve a filament with a diameter of 1.75 ± 0.05 mm, using an extrusion die with an orifice of 1.75 mm. The extruded filament was cooled down in a water basin to avoid sticking of the filaments. Other than extruded filaments, one commercial filament Ninjaflex TPU (SH) 85 A (NinjaTek, Fenner Precision Polymers, Lititz PA USA) was used in the study.

**Table 1:** Overview of TPU materials

Name	TPU type	Shore hardness in A
Elastollan 90A	Polyester-based	90
Elastollan 1190	Polyether-based	90
Elastollan 80A	Polyester-based	80
Elastollan 1180	Polyether-based	80
Ninjaflex	polyester-based	85

### 3D printing TPU template

The samples were designed as 20 × 20 × 10 mm cubes using Onshape 3D modeling software and Lulzbot Cura open-source slicing software. The samples were modeled as cellular structures with square cells in X–Y directions. The cell size was 2.5 mm, and the strut size was 150 microns. The cellular structures were then printed using a RepRap-class [38] open-source Lulzbot TAZ 6 (Fargo Additive Manufacturing, Fargo, ND, USA). For printing the samples, a nozzle with a diameter of 150 microns, a printing speed of 10 mm/s, a printing temperature of 240 °C and a bed temperature of 70 °C were used.

### Impregnation of 3D printed TPU structures with Durazane 1800

In first stage, each sample was first submerged to swell in a solution of 5 ml acetone and 500 µl Pt catalyst—platinum divinyltetramethyldisiloxane complex, ≈ Pt 2% in xylene (CAS number: 68478-92-2, Sigma-Aldrich, St. Louis, MO, USA) diluted to 0.1%. This resulted in the infiltration of PU structures with an acetone solution containing Pt catalyst. In the second stage, 5 ml polysilazane preceramic polymer (Durazane 1800; CAS: 503590-70-3; Merck, Darmstadt, Germany) was added to the solution. The role of the infiltrated Pt catalyst was to further promote the crosslinking of polysilazane via hydrosilylation reaction [39] between the Si–H and the C=C moieties present in the silicon polymer, within the PU structure. The samples were left soaking in the solution for 4 h, then taken out and dried in air for 24 h.

### Pyrolysis

The samples were pyrolyzed using an alumina tube furnace (GERO, Neuhausen, Germany) and nitrogen flow 300 ml min^−1^. A ramp of 2 °C min^−1^ was used up to 400 °C, including a dwell time of 3 h at 160 °C to ensure the crosslinking step. As found in TG, TPU was decomposed (− 95%) around 450 °C and ceramization began in this temperature region; accordingly, a slower ramp of 0.5 °C/min was used from 400 till 800 °C to ensure the structural integrity of cellular structures. Furthermore, the heating cycle continued at 2 °C/min up to 1200 °C with 1 h of holding time before natural cooling to room temperature.

### Density measurement

Skeletal density of the pyrolyzed cellular structures was measured with an He pycnometer (Ultrapyc 3000, Anton Paar), while the geometrical density (bulk density) was measured simply by the mass and volume of the samples.

### Thermogravimetric analysis, TGA

The decomposition of the different TPUs used in this study was checked by thermogravimetric analysis (TGA) (Jupiter F3 STA 449, NETZSCH, Selb, Germany) performed up to 700 °C with a heating rate of 5 °C/min under flowing nitrogen atmosphere (70 ml min^−1^). Using Netzsch Proteus software, the derivative of the TG signal was calculated and used, together with the total mass loss, to compare the thermal decomposition of the different TPU materials.

### Fourier transform infrared spectroscopy, FT-IR

The Fourier transform infrared (FTIR) spectroscopy was measured with a Tensor 27 instrument (Bruker Optik GMbH, Ettlingen, Germany). The solid samples were clamped under spring tension with the side to be analyzed on the ATR crystal. The samples were irradiated with polychromatic infrared radiation with the wavenumber range from 400 to 4000 cm^−1^. The recorded spectrum was used to identify the components by comparing the spectra with KnowItAll database [40].

### Scanning electron microscopy with energy dispersive X-ray spectroscopy, SEM–EDX

The EDX and SEM images were captured using a scanning electron microscope Jeol JSM-IT300LV (Jeol Ltd., Tokyo, Japan). The elemental composition was analyzed using EDX line scan across the cross section of a single strut of the ceramic samples.

### High-temperature stability of the ceramic samples

High-temperature stability of the ceramic lattices was evaluated by performing TGA experiments in inert (N_2_ flow, 50 ml min^−1^) and oxidative (air flow, 50 ml min^−1^) atmospheres. A Netzsch STA 409 apparatus (Netzsch Gmbh, Selb, Germany) was employed with a heating rate of 5 °C up to 1500 °C (N_2_) and 1200 °C (air). For the tests in N_2_, small fragments of the 3D structures were loaded in the alumina crucibles of the TGA apparatus, while for the tests in air, the ceramic lattices were previously ground to fine powders using an Agate mortar.

## Experimental results

### Characterization of TPU filaments: FT-IR study

The four types of TPU filaments—namely, polyester SH90A and SH80A, and polyether SH90A and SH80A—along with the commercial Ninjaflex, have been characterized by collecting FT-IR spectra.

**Fig. 1:**
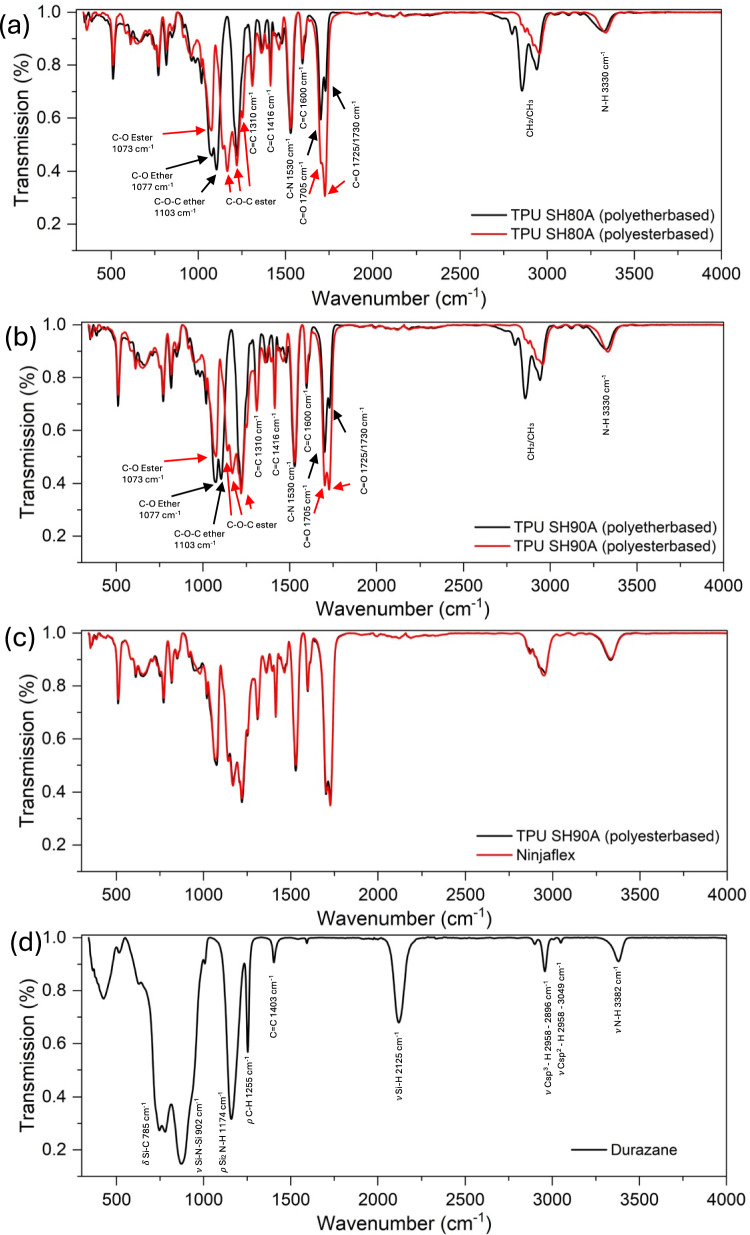
FT-IR spectra comparison of **a** ether- and ester-based TPU of shore hardness 80 A and **b** 90 A. **c** Similarity between polyester-based and Ninjaflex spectra. **d** Spectra of preceramic polymer Durazane 1800

The characteristic peaks of different TPUs and preceramic polymer are listed in Tables 2 and 3, respectively. Figure 1 shows the FT-IR spectra of the studied polyurethane and the Si-based Durazane 1800 precursor. Ether-based TPU can be recognized by the strong peak at 1103 cm^−1^ (C–O–C, ether), while the ester-based TPU shows a doublet at 1166 and 1143 cm^−1^ (C–O–C, ester) (Fig. 1a, b). Carbonyl (C = O) group belonging to the urethane functionality leads to a doublet at 1730–1705 cm^−1^ for the polyether-based TPU and at 1725–1705 cm^−1^ for the polyester-based TPU. According to Mailhot [41], the peak at lower energy, at 1705 cm^−1^, identifies C = O groups hydrogen bonded with N–H, while the peaks at higher energies, 1730–1725 cm^−1^, are associated with free C = O [42]. In ether-based TPU, the most intense signal corresponds to the C = O groups hydrogen-bonded with N–H at 1705 cm^−1^, whereas in ester-based TPU, the situation is reversed, with the most intense peak being that of the free C = O at 1730-/1725 cm^−1^. This can be rationalized knowing that the TPU structure phase separate into the hard segments (H)—formed by the urethane functionality—and the soft segment (S) containing the ether or ester bonds [41]. Accordingly, in ether-based TPUs, C = O moieties are present only within the urethane of the hard segments, making them prone to bond with the N–H donors of the same group. Conversely, in ester-based TPU, C = O bonds are also present in the soft segments. However, being farther away from the hard segments containing N–H groups, they have a lower probability of forming hydrogen bonds. For this reason, in ester-based TPUs, the most intense peak is due to free C = O groups, not H-bonded.

**Table 2:** FT-IR bands of the ester- and ether-based TPUs

Polyether TPU (80 SH)	Polyester TPU (80 SH)	Band	Intensity
Wavenumber (cm^−1^)			
3330	3330	N–H	Weak/weak
2940, 2850, 2800	2950, 2880	CH_2_/CH_3_	Medium/weak
1730, 1705	1725, 1705	C = O	Strong/strong
1600	1600	C = C	Medium/medium
1530	1530	C–N	Strong/strong
1416, 1310	1416, 1310	C = C	Weak/weak
	1252, 1222, 1166, 1143	C–O (ester)	Strong
1103		C–O–C (ether)	Strong
1077	1073	C–O	Strong/strong
1000–600	1000–600	C = O, CH_2_	Weak/weak

**Table 3:** FT-IR bands of Durazane pre-ceramic polymer

Wavenumber (cm^−1^)	Assignments
3382	ν N–H
3049	ν Csp^2^–H
2958–2896	ν Csp^3^–H
2125	ν Si–H
1403	C = C
1255	δ C–H (Si–CH_3_ deformation)
1174	ρ Si_2_N–H
902	ν Si–N–Si
785	δ Si–C

The FT-IR spectrum of commercial Ninjaflex TPU overlays with that of polyester-based TPU suggesting that Ninjaflex TPU is likely an ester-based TPU (Fig. 1c). Finally, the infrared analysis of commercial Durazane shows characteristic peaks at 3382 cm^−1^ (νN–H), 3049 cm^−1^ (Csp2–H), 2958 2896 cm^−1^ (Csp^3^–H), 2125 cm^−1^ (νSi–H), 1403 cm^−1^ (C = C), 1255 cm^−1^ (δSi–CH_3_), 1174 cm^−1^ (ρSi_2_N–H), 902 cm^−1^ (νSi–N–Si), 785 cm^−1^ (δSi–C) (Fig. 1d).

### Characterization of TPU filaments: TGA study

**Fig. 2:**
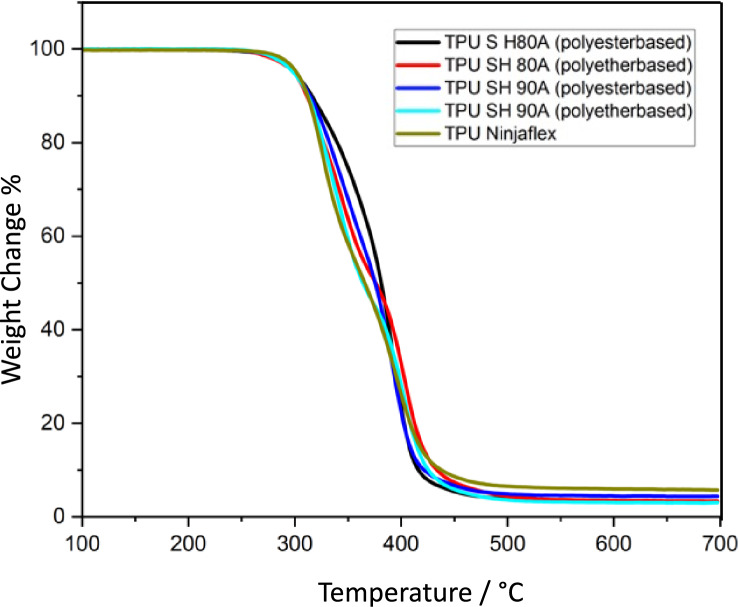
TGA curves of the starting TPUs recorded under Ar (or nitrogen) flow using heating rate 5 K min^−1^

Figure 2 displays TGA curves up to 700 °C. Beyond 500 °C, there is no further change in the weight of the samples. Accordingly, all TPUs decompose almost completely with a weight loss of ~ 95%, irrespectively from the composition (ester vs ether-based) and shore hardness (SH80A vs SH90A). The pyrolysis of TPU occurs in two consecutive weight loss steps: the first, in the range of 300–390 °C, is attributed to the decomposition of the hard segments, while the second, occurring between 390 °C and 450 °C, is associated with the decomposition of the soft segments [43].

### Characterization of the impregnation process: FT-IR study

**Fig. 3:**
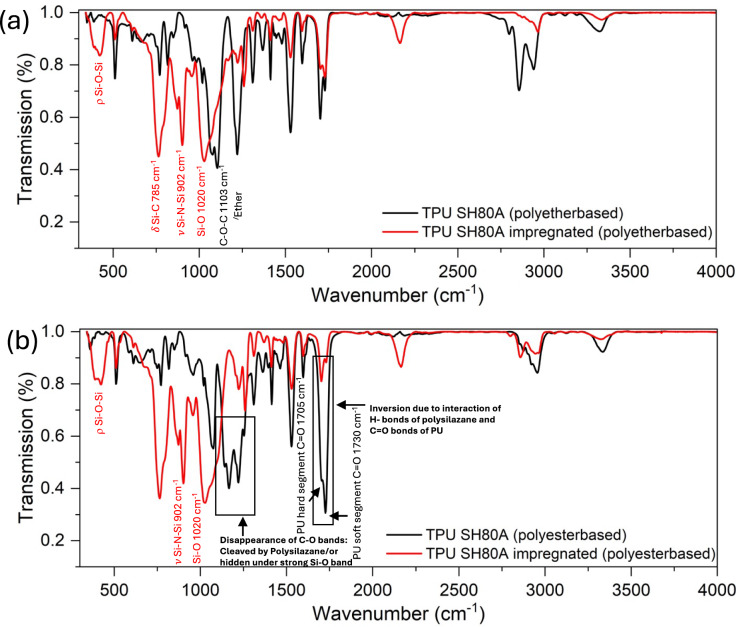
FT-IR spectra of the Durazane 1800-impregnated 3D structures compared with the neat TPUs: **a** polyether-, **b** polyester-based

To study the interaction occurring between the TPUs and the Durazane during the impregnation process, FT-IR spectra of the impregnated samples were compared with those of neat TPUs (Fig. 3a, b).

Notably, for both TPUs, the spectra primarily exhibit absorption peaks attributed to polysilazane, likely due to the enrichment of Durazane on the surface of the 3D-printed structure. The most intense peaks of the FT-IR spectra at approximately 902 and 785 cm⁻^1^ are attributed to N–Si–N and Si–C vibrations, respectively.

Furthermore, the peak observed at 1020 cm⁻^1^, with a shoulder at 1100 cm⁻^1^, suggests a partial oxidation of the Si–polymer (Si–O stretching), which is consistent with the appearance of a new wide absorption band around 450 cm⁻^1^, assigned to Si–O–Si rocking vibration.

The chemical interaction between Durazane and polyurethane primarily occurs through the formation of hydrogen bonds between the N–H moieties of polysilazane and the carbonyl groups of TPU. This interaction likely involves the hard segments (HS) containing the C = O bonds, and in the case of ester-based TPU, it may also involve the C = O groups within the soft segments.

Another type of interaction could involve the organic bonds, namely, the methyl groups, CH_3_, of the polysilazane and the CH_2_ groups of the soft segment. The Durazane-impregnated polyester-derived TPU shows an inversion of the intensity of the IR bands between the hydrogen-bonded (at 1705 cm^−1^) and the free C = O bonds (at 1730 cm^−1^) with a prevalence of the H-bonded C = O. This could be explained assuming that new N–H–O = C bonds are formed in the Durazane-impregnated sample. Another result, worth of notice, is the disappearance of the bands related to C–O (at 1143 and 1166 cm^−1^, polyester-based TPU). This evidence could suggest, on one hand, that ester groups are cleaved by the interaction with Durazane or, more simply, that the bands of the TPU are hidden under the strong band of Si–O at 1070 cm^−1^.

### Characterization of the impregnation process: volume swelling and mass increase

**Fig. 4:**
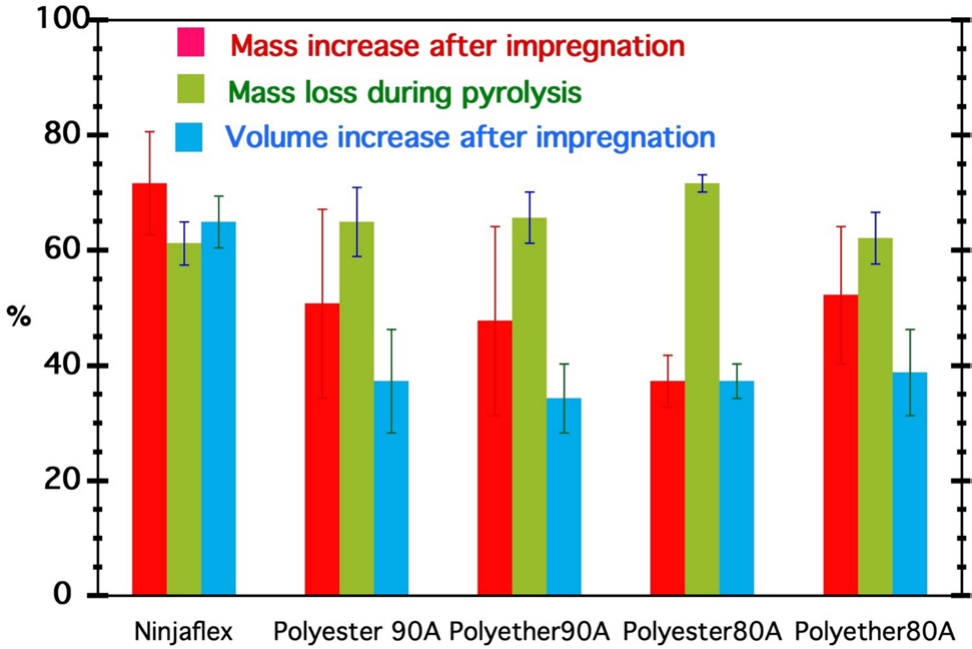
Mass increase of the 3D printed TPU structures after impregnation (red bars), mass loss during pyrolysis (green bars) and volume increase after impregnation (blue bars)

The efficiency of the impregnation process can be assessed by analyzing the mass and volume change associated with impregnation. Ninjaflex samples exhibit a remarkable 70% weight increase and 65% volume swelling after impregnation, and a subsequent 60% weight loss during pyrolysis. The pure ether- and ester-based TPUs show weight increases in the range of 35–50% and weight losses around 60–70% (Fig. 4). The variance in the figures can be attributed to environmental and processing factors. We observe that the pyrolysis weight loss increases when the mass gain during impregnation decreases. This can be rationalized as follows. Since polyurethane decomposes almost completely, with a weight loss of ~ 95%, the weight loss of the impregnated polyurethane structure is very high when the impregnation is not very efficient (resulting in low weight gain). Conversely, the ceramic yield is higher when the increase in weight after impregnation increases. Accordingly, the ceramic yield for the five studied samples is reported in Table 4.

**Table 4:** Ceramic yields of SiOC (N) produced with different TPUs

Sample	Ninjaflex	Polyester 90A	Polyether 90A	Polyester 80A	Polyether 80A
Ceramic yield (wt%)	39	35	34	29	38

The experimental results reported in Fig. 4 indicate the efficiency of the impregnation process. Considering the mass increase, the Polyether 80 A and Polyester 80 A show the highest and lowest efficiency, respectively, among all four TPU types. If the assessment is based on the volume increase, however, then all four TPU types show a comparable efficiency. In any case, Ninjaflex seems to be the best TPU both in terms of volumetric swelling and mass increase. As mentioned previously, Ninjaflex is a commercial product, based on polyester-based TPU (Fig. 2c). It is unfortunate that the material makeup is not available as the properties may be more easily optimized in the future. Based on our results, however, we can conclude that the higher volume increase must be related to other additives used in the Ninjaflex material. Future efforts to increase access to materials makeups could have far reaching benefits for both science and society [44]. In addition, all four TPUs do not reflect any tendency in mass/volume increase during impregnation and mass loss during pyrolysis, which suggests that the process is not related to the type or shore hardness of the studied TPU.

### Characterization of the ceramic cellular structures

#### Density, SEM and EDX

The skeletal density measured on the ceramic structure is reported in Table 5. The skeletal density of the ceramic struts is close to 2.00 g/cc, in line with previous results [36]. The density measured on the samples produced from Ninjaflex is slightly higher (2.08 g/cc, ca 4%) compared to the other composition. It could be due to the slightly higher mass uptake during impregnation. The bulk density ranges from 0.31 to 0.38 g/cc without suggesting any trend among the investigated samples.

**Table 5:** Skeletal and bulk density of the pyrolyzed ceramic cellular structures

Sample	Ninjaflex	Polyester 90A	Polyether 90A	Polyester 80A	Polyether 80A
Skeletal density (g/cc) ± StDev	2.08 ± 0.01	1.98 ± 0.06	1.99 ± 0.02	2.01 ± 0.02	2.01 ± 0.10
Bulk density (g/cc) ± StDev	0.31 ± 0.05	0.31 ± 0.07	0.37 ± 0.03	0.37 ± 0.03	0.38 ± 0.04

According to the literature [45, 46], the skeletal density of SiOC(N) ceramic samples pyrolyzed at 1200 °C and obtained using only the preceramic polymer (Durazane) in an inert atmosphere (Ar or N₂) ranges from 2.08 ± 0.12 to 2.416 g/cm^3^, indicating a rather high variability [47].

The values measured in the present study are slightly lower than the density values our group reported in previous publications [45, 46]. This result seems plausible, as the presence of TPU may increase the free carbon content and a higher amount of free carbon—which has a lower density than the SiOC(N) phase—is expected to result in a lower overall skeletal density [48].

Moreover, the skeletal density could tentatively be associated with the ceramic yield, assuming that a higher ceramic yield corresponds to a lower residual free carbon content derived from the decomposition of TPU. Accordingly, an increase in ceramic yield should lead to higher skeletal density. This trend is observed for the three TPU samples and the Ninjaflex sample (Fig. S2). The only composition that does not follow this trend is TPU Polyether 80A. In this case, additional experiments are needed to draw a definitive conclusion.

**Fig. 5:**
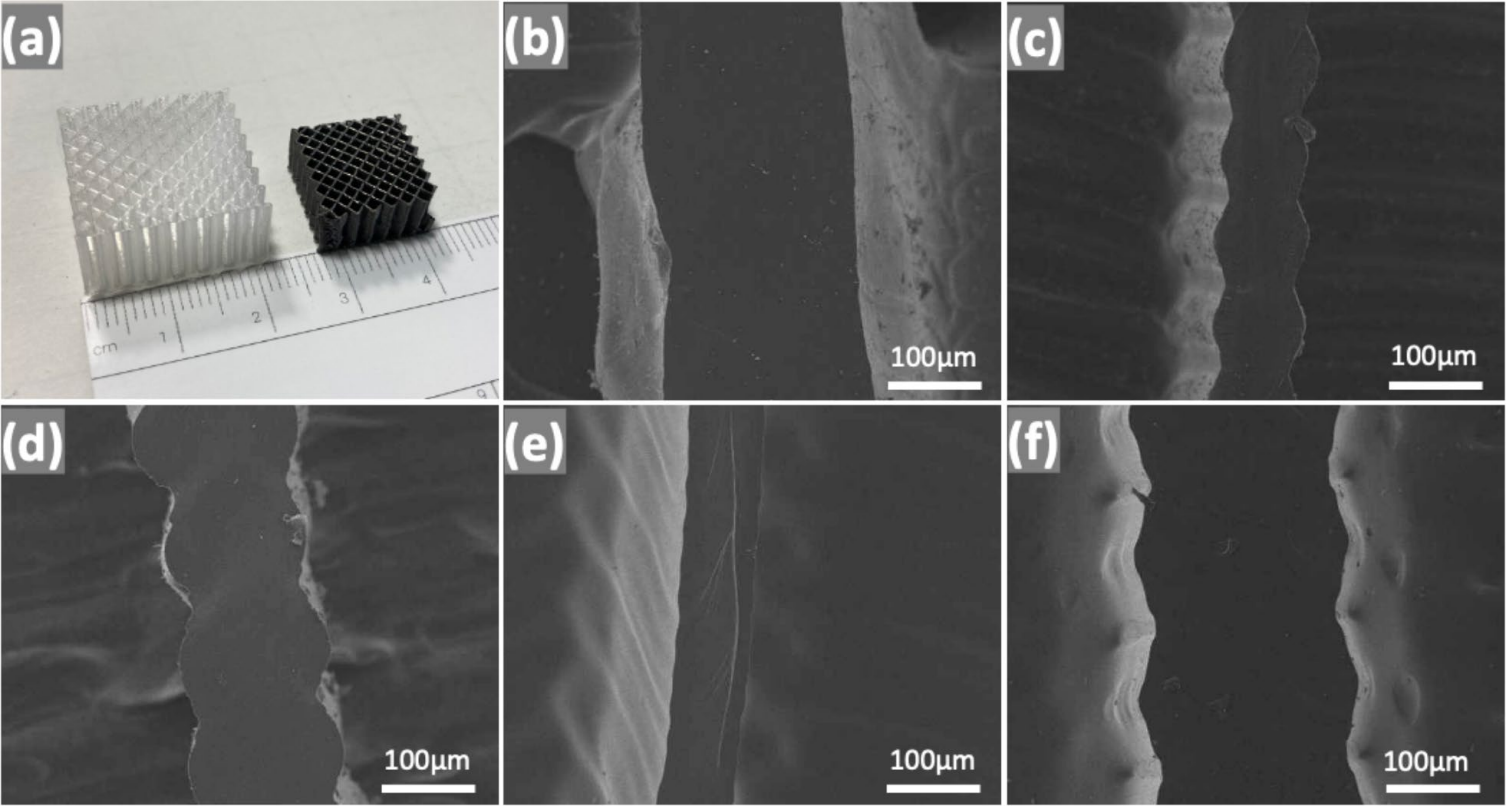
**a** Printed and pyrolyzed structure, and SEM of cross sections of the fractured struts, **b** Polyether 80 A, **c** Polyether 90 A, **d** Polyester 80 A, **e** Polyester 90 A, and **f** NinjaFlex 85A. It is worth noting that all the struts show no porosity and appear fully dense

A photo of the impregnated cellular structure and the corresponding ceramic one is shown in Fig. 5a. The fracture surfaces of the ceramic samples have been observed with the SEM. The SEM images presented in Fig. 5 confirm the absence of macroscale porosity, suggesting that Durazane successfully impregnated and swelled the TPU template. If the preceramic polymer had merely coated the surface of the polymer template without sufficient diffusion, the resulting ceramic struts would exhibit hollow cores, as demonstrated in previous studies [35]. To further investigate the potential presence of meso- or microporosity, we performed N₂ physisorption tests on two samples: Polyester 80 A and Polyether 80A. In both cases, the results did not indicate any significant porosity, allowing us to conclude that the ceramic samples are fully dense (Fig. S3). Furthermore, ceramic struts, of all the samples, have been found dense without any voids (see Fig. 5b–e), which indicates complete impregnation of Durazane in all TPU structures leading to dense struts after pyrolysis.

**Fig. 6:**
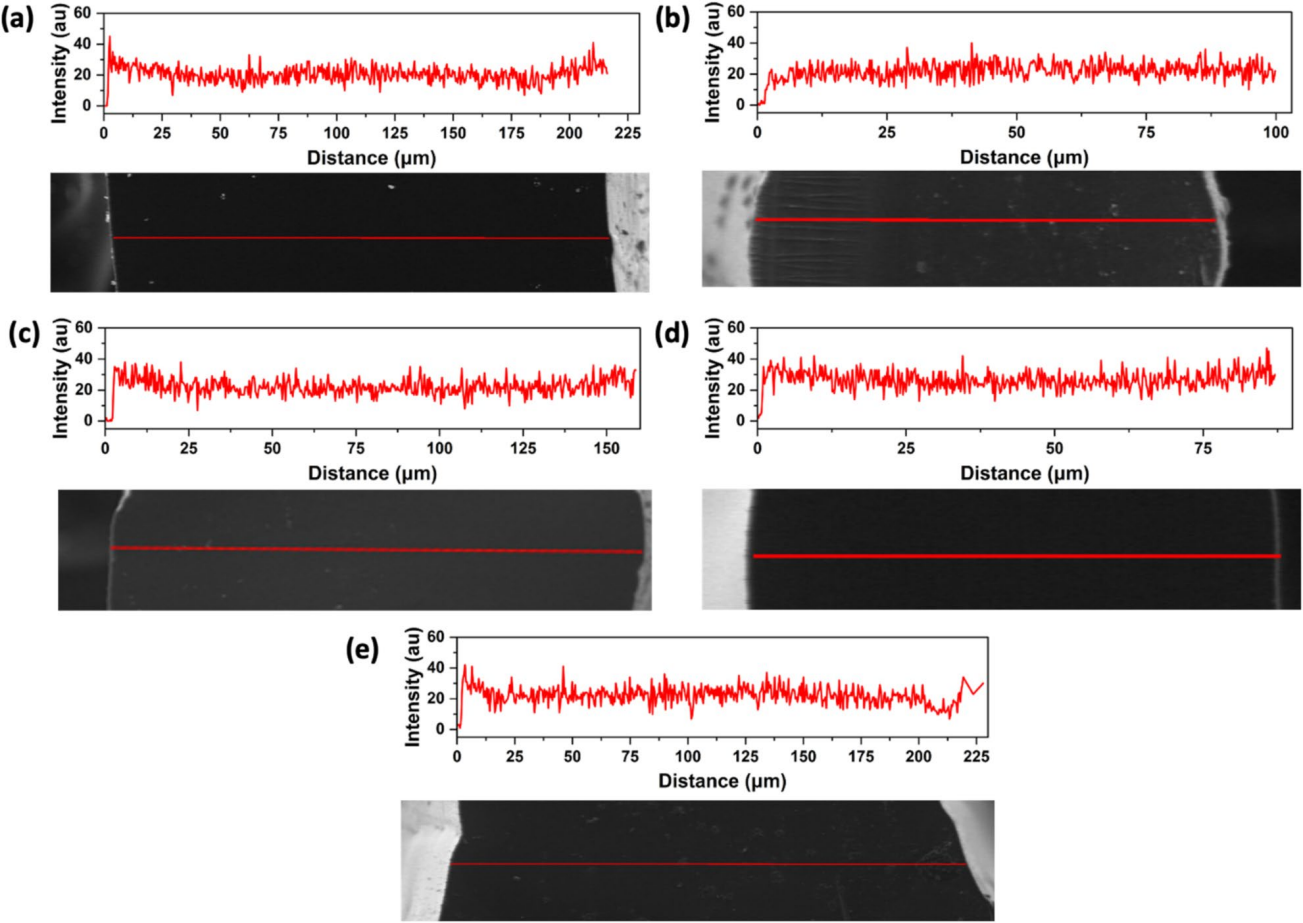
EDX line profile of Si for the cross section of **a** Polyether 80 A, **b** Polyether 90 A, **c** Polyester 80 A, **d** Polyester 90 A, and **e** NinjaFlex 85A

EDX line scans have been recorded to investigate the homogeneity of the chemical composition through the struts (Fig. 6). The distribution of Si, C, O, and N have been followed. Si is homogeneously distributed into the thickness of the struts, confirming the excellent diffusion of Durazane preceramic polymer through the volume of the TPU reaching the core of the struts.

#### High-temperature stability of the ceramic structures

**Fig. 7:**
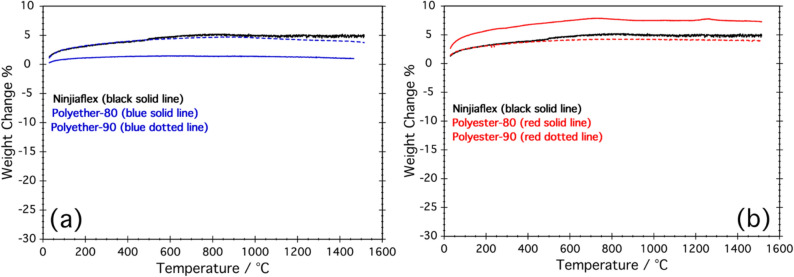
TGA curves recorded in N_2_ flow of the ceramic structures obtained using: **a** Ninjiaflex (black), Polyether 80 (blue solid line) and Polyether-90 (blue-dotted line); **b** Ninjiaflex (black), Polyesther 80 (blue solid line) and Polyesther-90 (blue-dotted line)

Figure 7 shows TGA recorded in N_2_ flow for the studied ceramic samples. TGA curves only show a slight initial weight increase assigned to the buoyancy effect and then the weight of all the samples is stable up to 1500 °C suggesting the excellent thermal stability of all the ceramics irrespectively from the used TPU.

**Fig. 8:**
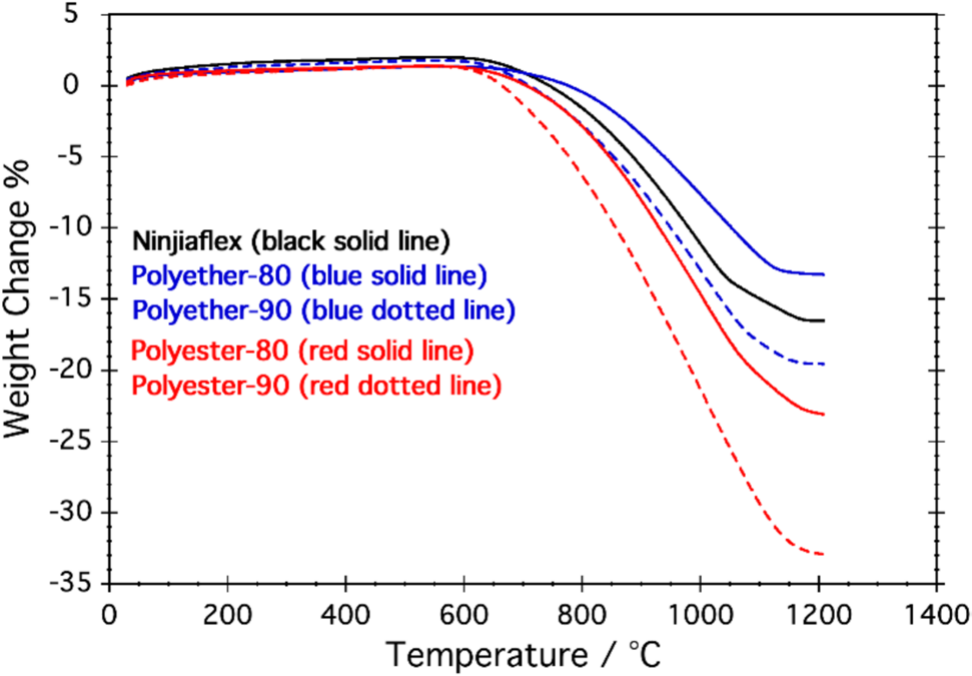
TGA curves recorded in air flow of the powdered ceramic structures obtained using: Ninjiaflex (black solid line); Polyether 80 (blue solid line); Polyether-90 (blue-dotted line); Polyester 80 (blue solid line) and Polyester-90 (blue-dotted line)

TGA obtained in airflow are reported in Fig. 8. All the samples undergo a weight loss from ca 600 to 1200 °C. This effect is due to the oxidation of the free carbon of the SiOC(N) ceramics [49]. Samples produced from Ninjaflex and polyether TPUs show a weight loss in the range 13–18%, while for polyester-based TPUs, the weight loss is higher, from ca 22% for polyesther-80 to ca 33% for polyester-90. These results seem to suggest that the free C content of the final ceramic varies from 13 to 33% and that polyester-based TPUs lead to a higher oxidation (more free carbon) compared with polyether-based ones. The explanation of these results is unclear and further experiments need to be done to rationalize this behavior.

## Conclusions

In this work, we fabricated cellular ceramic structures via 3D printing TPU structures, impregnation with polysilazane, and pyrolysis. The 3D printing was performed using FFF, while the ceramic was obtained through PDC process starting from a commercially available polysilazane, Durazane 1800.

We investigated the role of ester- and ether-based TPUs with two different Shore hardness (90A vs 80 A) on the impregnation of polysilazane. Regardless of the TPU type and Shore hardness, impregnation of the TPU 3D structure was successful and resulted in dense, non-hollow amorphous ceramic struts with similar skeletal densities. All polyester- and polyether-based TPUs showed a similar mass and volume increase after impregnation with high deviation. The mass loss during pyrolysis was also very similar for all the pure TPUs. These TPUs were then compared with one commercial TPU filament (Ninjaflex with a Shore hardness of 85 A). FTIR analysis showed that the commercial filament is based on a polyester-based TPU. Interestingly, this TPU showed a significantly higher volume and mass increase after impregnation and a slightly higher skeletal density. Several additives are used to modify TPU for specific applications. Based on our results, we assume that the commercial TPU filament has some additives that improve the swelling and the mass increase during impregnation with the PDC significantly. Future work is needed to identify this material and perform studies to optimize the volume fraction of the additive for this application. In conclusion, the study demonstrated that the combination of a polysilazane, like the commercial Durazane 1800 with thermoplastic polyurethane is ideal for the successful processing of cellular ceramic structures with dense struts using the FFF combined with the polymer pyrolysis method.

## Supplementary Information

Below is the link to the electronic supplementary material.

Supplementary file1 (DOCX 3812 KB)

## Data Availability

No datasets were generated or analysed during the current study.
