# Supplementary material for: Effect of thermoplastic polyurethane filament on the cellular ceramics structures obtained from material extrusion and polymer-derived ceramic
Source: Prog Addit Manuf. 2025 Jul 23;10(11):10331–42. doi: 10.1007/s40964-025-01243-w (PMC12537596; doi:10.1007/s40964-025-01243-w)
Supplement: Supplementary file 1 — Supplementary file1 (DOCX 3812 KB) [file 40964_2025_1243_MOESM1_ESM.docx]

**Supplementary information**

**Effect of Thermoplastic Polyurethane Filament on the Cellular Ceramics Structures Obtained from Material Extrusion and Polymer-Derived Ceramic**

**XRD**


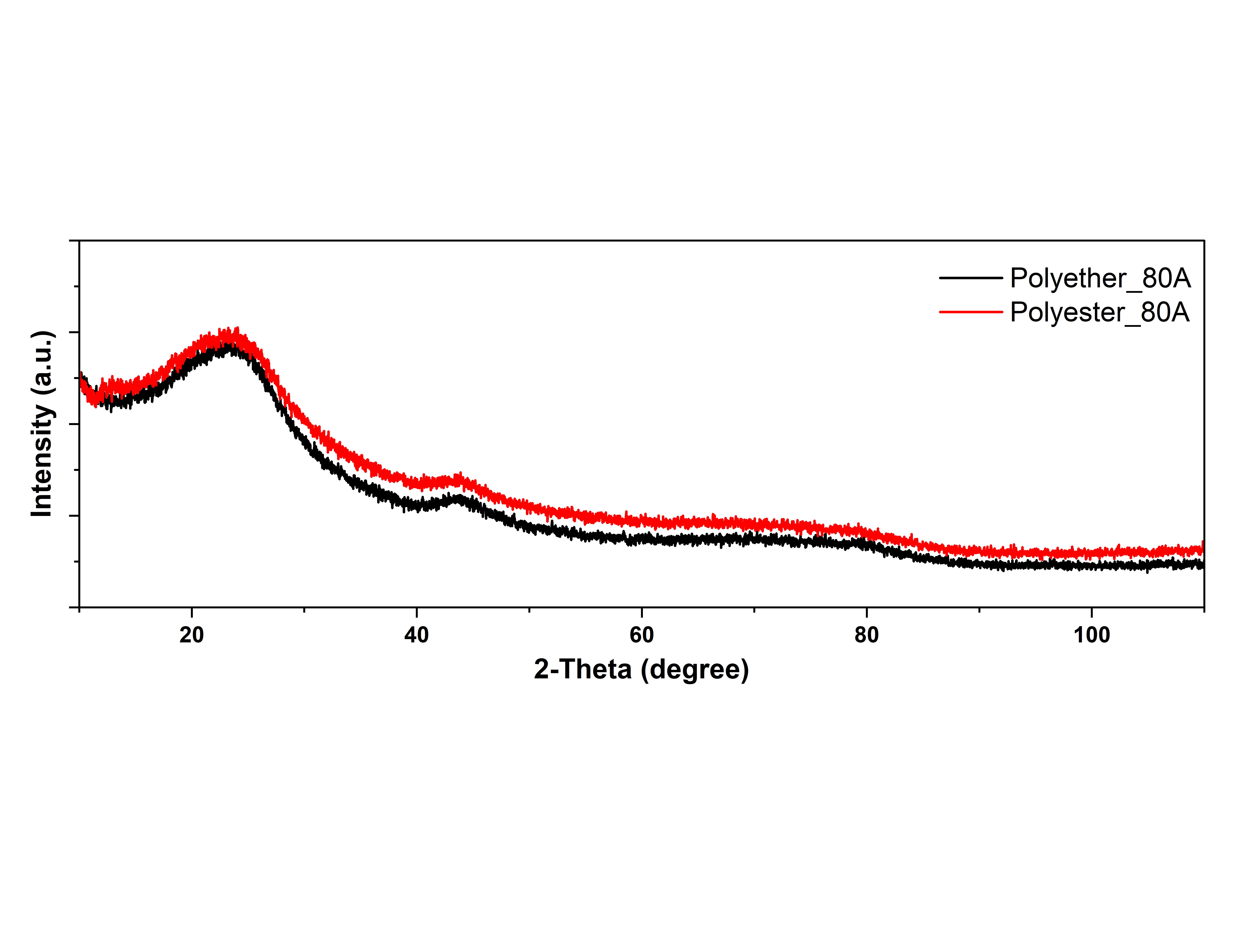


Figure S1 XRD of SiOC (N) produced with TPU (polyether 80A and polyester 80A) depicting only the amorphous phase without any crystallinity

**Ceramic yield vs skeleton density**

Figure S2 The plot depicts the relationship between the ceramic yield (wt%) and skeleton density

**BET**

**
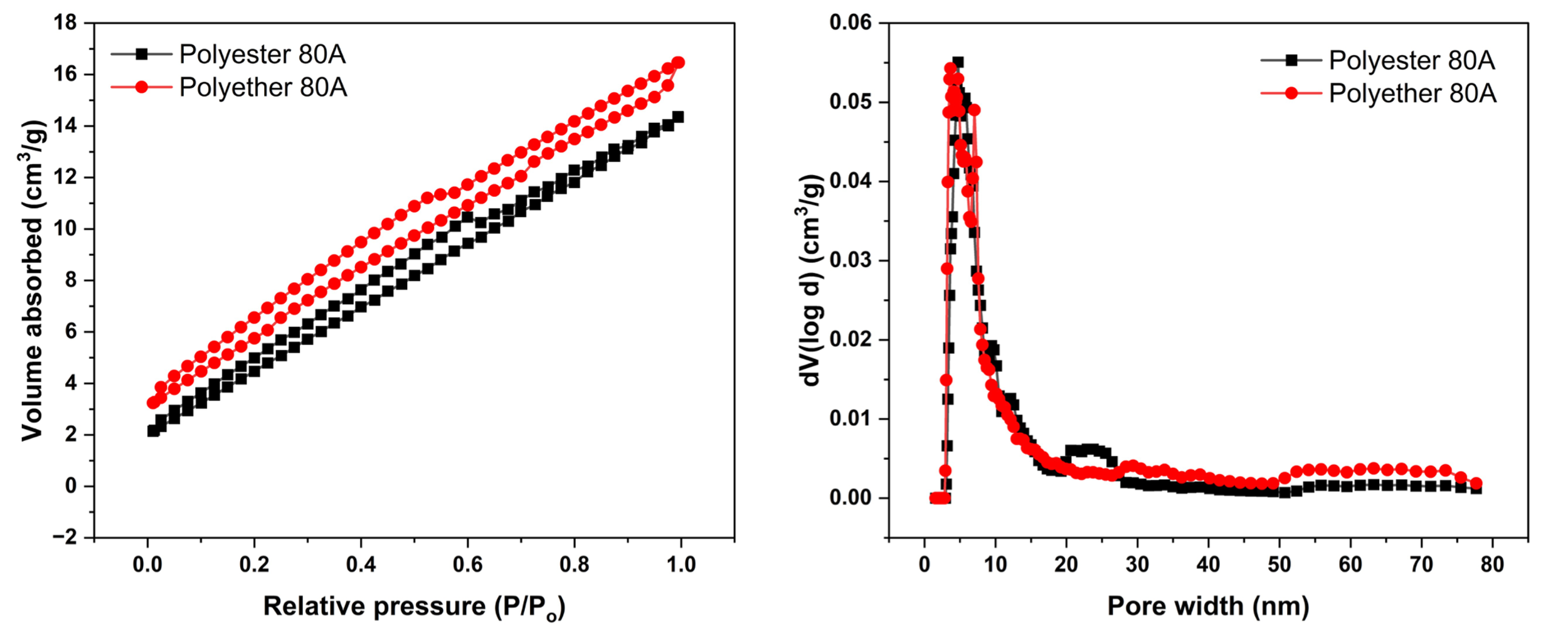
**

Figure S3 N_2_ adsorption-desorption isotherm and pore size distribution of the SiOC ceramic samples prepared with polyester 80A and polyether 80A
